# The Modulating Role of Self-Referential Stimuli and Processes in the Effect of Stress and Negative Emotion on Inhibition Processes in Borderline Personality Disorder: Proposition of a Model to Integrate the Self-Concept and Inhibition Processes

**DOI:** 10.3390/brainsci9040077

**Published:** 2019-03-30

**Authors:** Jean Gagnon, Joséphine Aldebert, Gasser Saleh, Wan Seo Kim

**Affiliations:** 1 Department of Psychology, University of Montreal, Montreal, QC H3C 3J7, Canada; josephine.aldebert@umontreal.ca (J.A.); gasser.saleh@umontreal.ca (G.S.); wan.seo.kim@umontreal.ca (W.S.K.); 2 Centre for Interdisciplinary Research in Rehabilitation of Greater Montreal (CRIR), Montreal, QC H2H 2N8, Canada; 3 Laboratoire d’Électrophysiologie en Neuroscience Sociale (LENS), Department of Psychology, University of Montreal, Montreal, QC H3C 3J7, Canada

**Keywords:** borderline personality, prepotent response inhibition, resistance to distractor interference, resistance to proactive interference, stress, emotion, self-concept, self-referential processes

## Abstract

Impulsivity is an important clinical and diagnostic feature of borderline personality disorder (BPD). Even though it has been reported that BPD individuals’ inhibition performance is significantly reduced in the context of negative emotion or stress, this literature shows mixed results, raising questions about the possible role played by other factors. Winter (2016) proposed that negative emotion stimuli can be more disruptive for BPD individuals’ attention control performance because they induce higher distractibility self-referential processes. This article aimed to systematically review the literature regarding the effect of stress and negative emotions on three main inhibition processes—prepotent response inhibition, resistance to distractor interference, and resistance to proactive interference—in BPD and to verify the putative modulating role of self-referential stimuli and processes on these inhibition processes. All English and French experimental studies published until August 2018 were searched in PsychINFO and PubMED databases. The following keywords were used: “borderline* AND inhibit* OR interference* OR forget* OR task* AND emotion* OR stress* OR affect*”. A total of 1215 articles were included in the study. After full text revision, twenty-six papers were selected for review. The results of this review indicate that when stimuli or procedures involve self-reference stimuli or processes, BPD individuals’ performance seems to be more disrupted in all three inhibition processes. A model based on Winter’s and Kernberg’s models is proposed with the aim of integrating the self-concept with inhibition processes in BPD.

## 1. Introduction

### 1.1. BPD and Impulsivity

Borderline personality disorder (BPD) is the most common personality disorder with estimated prevalence of 1.6%–5.9% in the general population [1] and 10%–20% among individuals receiving treatment in inpatient and outpatient clinics [1]. This costly mental disorder is challenging for clinicians as it alters the functioning of individuals at multiple levels. Indeed, BPD is characterized by extreme fluctuations in mood and sense of self, instable relationships, and marked impulsivity [1]. Impulsivity is a major diagnostic criterion and manifests itself in various high-risk behaviors, such as substance use, unprotected sex, reckless driving, temper outbursts, binge eating, and suicidal or self-damaging acts [1]. These behaviors have several consequences involving an undermining of their relationships, occupational functioning, and overall stability, but can also lead to serious legal and health problems as well as a risk of death. Therefore, because of the frequency and dangerousness of BPD impulsive behaviors (between 42.5% and 99.2% of individuals with BPD [2]), it is essential to understand what causes and maintains impulsivity in this population in order to intervene more effectively and to reduce its occurrence. Impulsivity is a multifaceted construct which has been assessed with self-report measures, behavioral laboratory measures, and event-related potentials (for a review see Bornovalova, Lejuez, Daughters, Rosenthal, and Lynch [3] and Gagnon [4]). In the last two decades, a growing interest has emerged in the use of behavioral tasks for studying the neuropsychological mechanisms associated with impulsive behaviors in order to clarify the nature of BPD’s impulsivity. There exist two groups of impulse control paradigms used in neuropsychological studies. Focusing on complex behavioral patterns, the first group is comprised of tasks assessing delay discounting and decision making. The delay discounting principle refers to the fact that the value of a reward is discounted as a function of the delay before receiving it. According to this principle, impulsive individuals prefer smaller immediate rewards over larger delayed rewards. In return, they prefer delayed larger negative consequences over immediate smaller negative consequences. In BPD patients, this preference could explain why they choose to engage in self-harm behaviors that give them immediate benefits of temporary emotional distraction and relief from intense negative affect, despite potential long-term negative consequences. Lawrence, Allen, and Chanen [5] administered a delay discounting task to BPD and control participants before and after a mood induction with the intention to verify if a change in mood would exacerbate the preference for an immediate reward. The results indicated that the BPD patients had a greater preference for immediate gratification and a higher rate of discounting the delayed reward than the controls. However, the rate of discounting did not change for the BPD group after mood induction whereas there was a change for controls, indicating less discounting of the delayed rewards following mood induction. In other words, controls shift toward greater self-control after their mood change whereas BPD patients did not, suggesting a failure to reduce impulsivity after rejection. The Iowa Gambling task is designed to study the integration of emotion and cognition in decision processes [6]. It simulates real-life decision making with uncertainty concerning premises and outcomes as well as rewards and punishments. Several studies have shown that BPD patients showed less advantageous choices on the Iowa Gambling task than did the healthy comparison subjects [7,8]. For example, BPD patients without a substance abuse disorder made fewer advantageous choices than controls [7]. However, other studies showed that BPD patients have normal performance on the Iowa Gambling task [9,10].

The second group of performance tasks used in neuropsychological studies assesses inhibition-related functions. The ability to inhibit a response and the capacity to take into account the future consequences of action can act conjointly to promote impulsive behaviors. For example, a poor ability to inhibit a prepotent response in emotional context predicts disadvantageous choices and risky decisions [11]. The present study will focus on inhibition processes underlying BPD impulsivity.

### 1.2. BPD and Inhibition Processes: The Role of Stress and Negative Emotions

Inhibition processes, which enable one to perform goal-directed behaviors, have been described as playing an important role in the occurrence of impulsive behaviors. From a neuropsychological point of view, impulsive behavior is viewed as a consequence of an inhibitory control failure [12,13,14]. Empirical research has demonstrated that inhibition control is not a unitary construct [14,15,16,17,18], and inhibition processes have been recently categorized according to the following three cognitive processes: prepotent response inhibition, resistance to distractor interference, and resistance to proactive interference [19,20,21,22,23].

Prepotent response inhibition (PRI) is defined as the ability to deliberately suppress dominant, automatic, or prepotent responses [22]. Among tasks assessing this cognitive process, the most frequently used are the go/no-Go (GNG) [24] and the stop-signal task [25] or sometimes called go/stop task [21]). The GNG consists of categorizing stimuli and suppressing a previously reinforced response. The stop-signal task consists of categorizing stimuli and withholding the learned response on some trials. Resistance to distractor interference (RDI) is the ability to resist or resolve interference from information in the external environment that is irrelevant to the task at hand [22]. The Emotional Stroop Task (EST) [26] is sometimes considered a PRI since the participants have to respond to the color in which color words and other words are printed and ignore the dominant tendency to read the words [22]. However, a recent study [18] indicated that this task is relevant to both PRI and RDI processes. Resistance to proactive interference (RPI) refers to the ability to resist memory intrusions from information that was previously relevant to the task but has since become irrelevant [22]. This process is often measured with the Direct Forgetting Task (DFT) [27], in which participants are exposed to several types of words and are cued to either remember or forget the presented words.

Several studies have demonstrated basic response inhibition deficits with emotionally neutral stimuli or conditions [16,28,29] among BPD individuals compared to healthy controls (HC) while other studies did not support such an observation [7,30,31,32,33,34,35,36]. A review of literature on different inhibition processes in BPD [17] indicated that participants’ “cold” impulse control towards non emotional stimuli is less impaired than their “hot” impulse control, i.e., components involving emotional and motivational aspects, which led the authors to conclude that BPD impulsivity might be secondary to an emotional dysregulation. Moreover, since impulsive behaviors of BPD individuals often take place in stressful or negative emotional contexts [37,38], and because emotional hyperarousal has frequently been described as a crucial factor that interferes with goal-directed behavior [39,40] and thus contributes to impulsiveness, the most recent studies of this line of research have investigated the interaction between stress, negative emotion and inhibition control. To do so, the authors either used various procedures to induce stress or a negative emotional state in participants before the completion of the inhibition task, or used various kinds of emotional stimuli during the task. However, results of these studies remain mixed. Among studies assessing PRI during a stressful [41,42,43] or negative emotional context [33,44,45,46], some reported that BPD participants performed worse than the HC [42,45,46], but this observation has been inconsistently supported [33,41,43,44]. With regards to studies assessing RDI via the EST task, Kaiser, Jacob, Domes, and Arntz’s [47] meta-analysis study concluded that some negative emotional stimuli, such as BPD-specific/personally relevant stimuli, disrupt BPD individuals’ performance more than the rest, suggesting that the performance can be varied depending on characteristics of stimuli. Finally, among the few studies which assessed RPI in a negative emotional context, one study reported deficits in BPD participants [48], while another did not support this observation [49].

### 1.3. BPD and Inhibition Processes: The Role of Self-Referential Stimuli and Processes

In her review of studies on attention control towards emotional stimuli in BPD individuals, Winter [50] reported heterogeneous evidence from behavioral tasks due to methodological differences between studies. More importantly, she pointed out the lack of control across studies over psychological factors that can play a role in modulating the attention–emotion interaction, i.e., the way emotional stimuli are processed. One of these factors concerns self-referential processes which refer to the preference for people to process information that has personal relevance. In congruence with their negative self-concept, the author rightfully suggests that negative stimuli can be more disruptive for BPD participants’ control attention simply because they induce among them a more pronounced self-referential process than positive or neutral stimuli. In conjunction with their interpersonal difficulties that they frequently experienced and their repetitive aversive childhood experiences, Winter proposes that BPD participants would be more reactive to social stimuli and trauma-related stimuli for similar reasons. In brief, the author’s hypothesis suggests that negative, social, or trauma-relevant information have the potential to capture more attention in BPD participants than controls because it involves a reference to the self-concept of BPD participants, resulting in a reduced resistance to distraction towards emotional stimuli. Although interesting, this hypothesis has not been empirically tested experimentally or via a systematic review.

Attentional control is required during goal-pursuit tasks since it is conceptualized as a filter of information [51]. It is proposed that the attention that is captured by emotional stimuli (or by stressful or emotional context) helps the individual to select a response to potentially relevant environmental cues but can also be detrimental to the functioning of inhibition processes that need cognitive resources [40]. Winter reviewed tasks in which participants have to use selective attention to goal-relevant stimuli in order to avoid distraction, which is consistent with the definition of RDI. Thereby, Winter’s review raises the question if the modulating effect of self-referential processes on RDI in BPD individuals can be extended to PRI and RPI.

There is some data supporting the view that BPD participants’ inhibition performance is diminished when stimuli or procedures are related to their self-concept. In their study, Ernst and collaborators [52] have exposed depressive subjects with or without BPD and HCs to a Cyberball task, a procedure involving inclusion or social exclusion, before testing them on a GNG task with neutral stimuli. Subjects with BPD showed inhibition performance impairments in both conditions compared to HC. Also, there was a significant correlation between the total of commission errors and so-called personality structural deficits assessed by the Borderline Personality Inventory [53]. More importantly, this correlation remained significant after controlling for the level of childhood trauma, which suggests, according to the authors, that “potentially traumatizing experiences have to meet with precarious psychic structures (which BPD is organized around) in order to put forth response inhibition deficits.” Another study reported consistent findings with a more direct measurement of identity disturbance. In order to evaluate the role of identity disturbance on impulsivity, Lowmaster [54] manipulated the coherence of the self-concept of high and low borderline personality (BP) features participants. For this purpose, she asked them to describe their “true self” by using five words (for details about this procedure, see Lowmaster [54]), a procedure intended to induce a stress on their self-concept coherence. Indeed, the high borderline features participants reported greater difficulty generating words to describe themselves compared to low BP feature participants. After completion of the self-definition task, participants were submitted to a go/stop task with neutral stimuli. Participants with high BP features had more difficulty in inhibiting their responses following the manipulation of self-concept coherence compared to participants with low borderline features. Therefore, the latter studies give some empirical support to the proposition that the interaction between negative emotional or stressful stimuli and PRI could be moderated by the processing of self-referential stimuli. Although self-reference processes should be taken into consideration when studying BPD inhibition, the role of these processes has not been examined up to now.

### 1.4. Winter’s and Kernberg’s Models

To help elucidate the specific aspects of inhibition (i.e., PRI, RDI, and RPI) in relation to problems of self-concept and emotion regulation, results of the present review will be examined against Winter’s and Kernerg’s models.

According to Winter’s model, the negative self-concept of BPD individuals disturbs attentional control by filtering negative information. This attention filter influences the information encoded in the working memory and consequently the information that is stored in long term memory. In turn, the information stored in long term memory modulates the processing of emotional information with a bias toward negative information. Although very interesting, this model has four limitations.

Firstly, this model does not take into consideration the influence of social aspects on inhibition processes while BPD impulsive behaviors take place mostly in social contexts [55], and BPD inhibition performance is often disrupted by social stimuli (for instance, Ernst and collaborators [52], Bertsch and collaborators [56], Dixon-Gordon, Tull, Hackel, and Gratz [57]). Secondly, the model does not explain the effect of the intensity of emotion stimuli [31,42] and in which way the emotional intensity is related with the self-concept. Thirdly, the model strictly focuses on the self-concept content neglecting the self-concept cohesion. It is known that BPD individuals suffer from an unstable self-concept and a lack of cohesion across social interactions [1]. Moreover, it has been shown that response inhibition performance in BPD individuals is reduced compared to HCs when the self-concept cohesion is stressed [54]. Finally, the model has been developed to explain RDI only whereas the results of the present review suggest that self-referential stimuli or procedures seem to impact also on the two other inhibition processes, PRI and RPI.

Kernberg’s [58] model of personality could be used to complement Winter’s cognitive model. Indeed, Kernberg’s conceptualization of BPD personality takes into consideration the self-concept in relation with the concept of the other, and the influence of emotional as well as cognitive aspects of these concepts on BPD individuals’ emotional and behavioral responses. More specifically, in Kernberg’s model, the self-concept is conceptualized as a representation of the self and a representation of another person linked by a positive or negative affect. So, the self-concept is intrinsically linked to the concept of others through an emotional tone, and influences the subjective experience and behavior in social interactions. In BPD individuals, the self-concept is viewed as organized at the so-called borderline level of personality which is characterized by a lack of integration between negative and positive segments of self- and other-representations. As a consequence, this lack of integration between polarized representations reduces the neutralization of aggressive affect disposition and increases the intensity of negative emotions. Moreover, this lack of integration within the self-concept deprives the individual from cognitive resources available to use adaptive defense mechanisms and self-control. Consequently, the BPD self-concept is related to the defense mechanism of splitting characterized by an involuntary effort in keeping actively separated the negative self- and other-representations from the positive self- and other-representations. The use of splitting results in sudden changes in mood and behavior. Given that Kernberg’s model accounts for the social stimuli, the intensity of emotion and the self-concept cohesion, it complements Winter’s model in the understanding of the relation between inhibition processes and self-referential stimuli and processes.

### 1.5. Objective of the Study

In summary, the mixed results obtained by studies on the effect of negative emotion and stress on BPD inhibition processes raise the question whether self-referential stimuli or processes modulate all three cognitive processes related to inhibition: PRI, RDI, and RPI. To the best of our knowledge, there has not been a systematic review covering these three processes with regards to the self-concept. This article aims to systematically review the literature regarding the effect of stress and negative emotion on these three main inhibition processes in BPD patients and to better understand the role played by self-referential processes on them. Since previous research did not always take into account the type of inhibition process that was involved in their behavioral tasks to measure impulsivity, results of the studies have first been analyzed according to the type of inhibition process involved in the task. Then, the results were categorized according to stimuli or procedures that potentially induced self-referential processes among participants before or during the completion of the behavioral task. The first goal of the present study was to verify if stress and negative emotional context or negative stimuli disrupt BPD performance on tasks measuring the three inhibition processes. The second goal was to verify if self-referential stimuli or processes impact on BPD’s inhibition performance during stress and negative emotional context or stimuli and if this impact was similar across the three inhibition processes.

We used a systematic approach to review the literature. One difficulty encountered was that neuropsychological studies used a variety of behavioral tasks to measure inhibition functions. Since methodological differences among studies could lead to the assessment of different aspects of impulsivity [3,13], it was important to make use of a recognized definition of inhibition in order to select studies. To do so, we used Friedman and Miyake’s [22] definitions of PRI, RDI, and RPI since they are largely recognized [18,19]. Another difficulty was to categorize studies according to the presence or absence of self-referential processes in the various stimuli or procedure they used to induce negative emotion or stress. To do so, we used Winter’s [50] criteria for BPD self-referential stimuli, to which we added Lowmaster’s [54] procedure to stress the cohesion of BPD individuals’ self-concept.

## 2. Materials and Methods

### 2.1. Search Strategy and Selection of Studies

**Figure 1:**
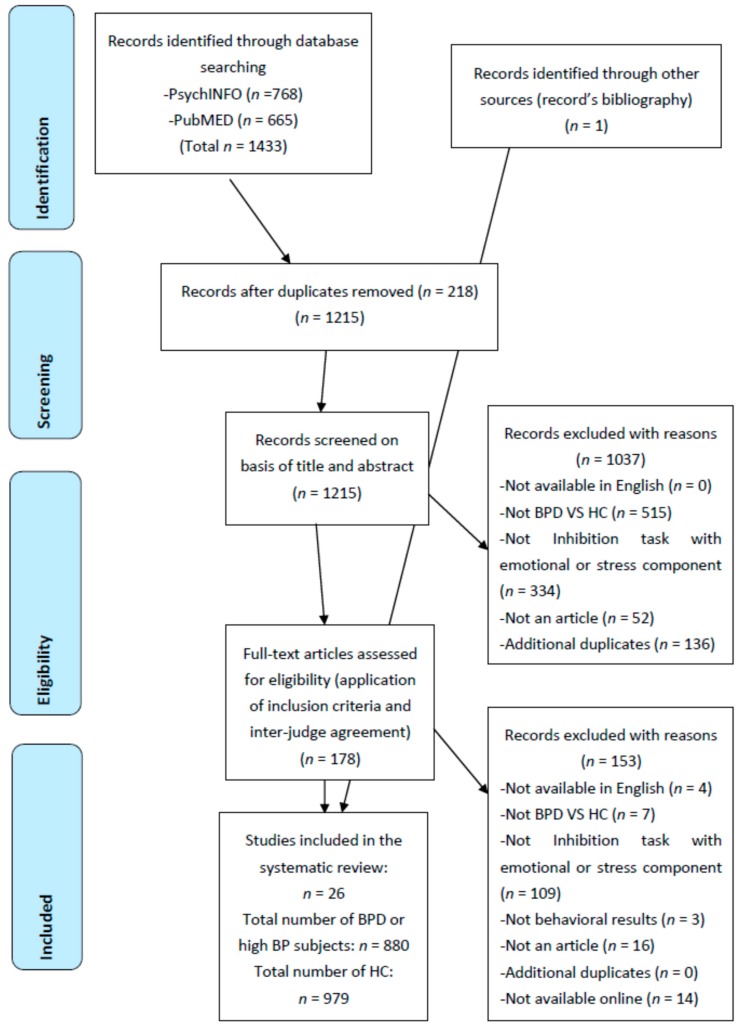
Preferred Reporting Items for Systematic Review and Meta-Analyses (PRISMA) flow diagram of the selection of studies.

The search strategy was based on the PRISMA (Preferred Reporting Items for Systematic Review and Meta-Analyses) guidelines [59] as illustrated in Figure 1. Given their importance in the literature of psychiatry and psychology, we chose PsychINFO and PubMed as a bibliographic database to search for articles and theses published up to August 2018. The following keywords were used; “borderline* AND inhibit* OR interference* OR forget* OR task* AND emotion* OR stress* OR affect*”.

A total of 1215 papers were found in the initial selection after eliminating duplicates (see the Figure 1 below). A second selection of studies was based on the title and the abstract which resulted in 178 studies. Then, the full text of the studies was screened and studies that did not meet inclusion criteria were discarded. During that screening, we added one article to our review based on a bibliographical record. Moreover, we complemented our database with studies reviewed by Kaiser et al. [47] in their meta-analysis on emotional bias for emotional stimuli in BPD except for two—one German article and one unpublished article. During the screening, an agreement between two of the authors (J.A. & J.G.) was reached when necessary in order to categorize less standard inhibition tasks into the proper cognitive process category, leading to 26 articles. Finally, a second verification of the 178 studies was made by the one author (J.A.), but this last screening did not lead to any additional study.

### 2.2. Inclusion and Exclusion Criteria

#### 2.2.1. Studies

Only experimental studies were included in our database whereas other studies (meta-analysis, case report, systematic and nonsystematic studies) on the same topic were full-text analyzed in order to find other records. The literature review was restricted to English and French language papers or theses published and accessible online.

#### 2.2.2. Behavioral Tasks

To be included, tasks had to assess a cognitive process that conforms to Friedman & Miyake’s [22] definition of one of the three inhibition processes as discussed above—PRI, RDI, or RPI.

Among studies assessing PRI, we selected five studies with GNG or a variation of the GNG task [33,43,45,52,60] and three studies with a Go/Stop task [31,46,54]. We also included two studies which comprised two Immediate Memory Tasks (IMT; [20,41,46]) and one Delayed Memory Task (DMT) [20,41]. Finally, we selected five studies with behavioral tasks consisting of response learning in which participants had to withhold a prepotent learned response [42,44,56,57,61]. We did not include tasks measuring participants’ allocation of attentional resources only, such as the Dot Prob Task [62] or the Dual Task [63].

Among studies assessing RDI, we selected nine studies with EST [49,64,65,66,67,68,69,70,71]. We also selected two studies with tasks that were in accordance with RDI’s definition, such as the Scrambled Sentences Test (SST) [72], in which participants were asked to rearrange six-word strings in order to create 5-word sentences by choosing and omitting one word [73] or to perform a verbal learning and interference task [74]. Each task requires from participants to ignore a distractor. We did not include studies that measured the effect of emotion on memory by manipulating the affective content of the story to be remembered. Finally, we selected two studies that measured RPI [48,49].

Furthermore, to be included, studies had to report behavioral results since this information was used to determine the presence or absence of inhibition deficits. For example, we excluded studies that reported only fMRI results.

#### 2.2.3. Emotional or Stressful Context

We selected tasks in which stressful or emotional stimuli or procedures were presented before or during the task. As mentioned above, we used emotion* OR stress* OR affect* in order to find the largest range of stress and emotion stimuli and procedures.

#### 2.2.4. Subjects

We selected studies in which BPD was defined according to a clinical diagnostic system such as the DSM [1] or the International Personality Disorder Examination (IPE) [75] or via a measure of BP features or borderline personality organization (i.e., Personality Assessment Inventory Questionnaire-Borderline—PAI-BOR [76]). Several studies used a measure of BP features or borderline personality organization because of the interest the nonclinical population represents for correlational studies [77] and the similarities observed between individuals with high BP features and individuals with a clinical BPD diagnostic in terms of etiology, symptomatology and level of functioning [78].

Since inhibition is an executive function that develops very early in life and continues to evolve into adulthood [79], we selected studies with adult participants only. No study was excluded on the basis of this criterion.

Finally, to be included, studies had to have a HC group in comparison to a BPD group. As the majority of studies did not have a clinical control group, this specification was not selected as a criterion. We excluded case studies, studies assessing BPD patients only, or BPD participants with a noncontrolled comorbidity.

### 2.3. Categorization of the Studies According to Self-Reference

As mentioned above, Winter [50] argued that stimuli which contain negative-, social-, or trauma-relevant information may elicit particularly strong self-referential processes in BPD participants. Even though general negative stimuli might be able to do so because of BPD participant’s negative self-concept, those stimuli per se can be viewed as less directly connected with the self-concept because of their less obvious social nature (i.e., negative words such as war or disease). Consequently, we chose to categorize stimuli or procedure as being able to induce self-referential processes if they contained negative social or trauma-relevant information (as opposed to nonsocial or nontrauma information).

Even though she recognized the instability of BPD self-descriptors and attitudes toward the self, Winter [50] did not address this issue in her study on attention–emotion interactions. This instability and lack of self-coherence was taken into account in Lowmaster’s [54] study in which the participants had to describe their “true self”. We thereby categorized self-descriptors stimuli and Lowmaster’s procedure [54] as self-referential stimuli and procedure. We also categorized words related to BPD specific cognitive bias, distorted beliefs, or schemas as self-referential stimuli.

In some studies, emotional stimuli were individually selected for each participant after a brief interview, in which, for example, they were asked about a personal event that invoked a negative emotion. In some cases, authors used the interview itself as the emotional procedure. These stimuli or procedures were qualified as “personally relevant” and were categorized as self-referential.

Finally, we chose to include negative valence stimuli or procedures and exclude positive valence stimuli or procedures in order to manipulate the self-referential factor while controlling for the emotion valence of stimuli.

All things considered, we categorized stimuli or procedures as being able to induce self-reference processes if they contained negative social information, negative trauma-relevant information, negative self-descriptors, procedures consisting in the description of oneself, negative stimuli related to BPD specific cognitive bias, distorted beliefs or schemas, and finally, negative stimuli or procedures which were personally relevant for the participant. Table 1 and Table 2 contain all studies categorized according to these criteria.

## 3. Results

Our literature review resulted in 26 studies that measured at least one of the inhibition processes among BPD participants in a negative emotional or stressful context. These 26 studies totalized 41 tasks involving different kinds of emotional or stressful stimuli or procedures. For example, Arntz, Appels, and Sieswerda’s [64] study included four kinds of negative emotional words in a EST. Carvahlo et al.’s [43] study and Winter et al.’s [71] study included two kinds of emotional procedures—one before and one during the inhibition task whereas some other studies included two inhibition tasks [41,46,49].

### 3.1. The Role of Stress or Negative Emotion in each Inhibition Dimension

The first aim of our study was to verify whether negative emotion and stress were consistently able to induce deficits in each inhibitory process in BPD participants. Among the 17 tasks assessing PRI, nine resulted in a decreased performance among BPD participants compared to HCs whereas eight tasks did not result in a group difference. Regarding the 21 tasks assessing RDI, 13 resulted in a decreased performance among BPD participants compared to the HCs whereas eight tasks did not result in a group difference. It should be noted that almost all RDI studies used EST except for two. Finally, among the three tasks assessing RPI, only one resulted in a decreased performance among BPD participants compared to HCs.

### 3.2. The Role of Self-Referential Processes in Modulating the Link between BPD Participants’ Inhibition Performance and Negative Emotional or Stressful Context

The second aim of this study was to verify if the presence of self-reference processes in tasks were influencing the BPD individuals’ inhibition performance. We divided the 41 tasks into two tables according to the presence (see Table 1) or absence (see Table 2) of a self-referential component in the emotional or stressful stimuli or procedure. Some studies are presented in both tables since they used both stimuli and procedures.

#### 3.2.1. Studies that Involved Self-Reference Processes

This category includes 19 studies totalizing 24 inhibition tasks (see Table 1). Among the nine tasks assessing PRI in which there were self-reference stimuli or processes, six resulted in a decreased performance among BPD participants compared to HCs whereas three did not result in a group difference. Regarding the 14 tasks assessing RDI interference with self-reference stimuli or processes, 10 resulted in decreased performance among BPD participants compared to HCs, whereas four did not result in a group difference. Finally, there was only one task assessing RPI with self-reference stimuli or processes and it resulted in decreased performance among BPD participants compared to HCs.

##### Stimuli or Procedures Containing Social Information

Carvahlo et al. [43] reported no group difference on a GNG task with fearful faces in the placebo situation (without induction of hydrocortisone before the task). On the contrary, compared to the HC group, Bertsch et al. [56] reported that the anger-prone BPD group was faster to approach than to avoid angry faces. In their study, Jacob et al. [33] used a short story telling a conflict between two friends in order to induce anger before a go/stop task but this procedure resulted in no group difference. On the contrary, Ernst et al. [52] reported that, compared to HC and MDD groups, the BPD-MDD group performs significantly worse at the GNG task after the exclusion situation. In the study of Paret, Hoesterey, Kleindienst, and Schmahl [61], participants were exposed to aversive social stimuli (aversive scene depicting human beings) and neutral stimuli during an operant conditioning task. The authors reported no group interaction between the kind of stimuli and task performance. In the study of Dixon Gordon, Tull, Hackel, and Gratz [57], the BPD group who performed a reinforcement learning task after a negative emotion induction (via the record of a personal narrative of a recent distressing interpersonal interaction with a close friend or family member about whom the participant felt “very upset”) showed a significant decrease in learning accuracy compared to HCs. Finally, with words referring to a negative view of others, Arntz, Appels, and Sieswerda’s [64] procedure produced more interference among BPD group than the HC group but did not produce more interference than other types of words (sexual abuse-related, negative self-descriptors, and general negative words unrelated to BPD pathology).

##### Stimuli or Procedures Containing Trauma-Related Information

Words referring to sexual abuse in the study of Arntz, Appels, and Sieswerda [64] produced more interference among the BPD group than the HC group but did not produce more interference than the other types of words (negative view of others, negative self-descriptors, and general negative words unrelated to BPD pathology).

##### Stimuli or Procedures Containing Mainly Self-Descriptors or Involving the Description of Oneself

The affective GNG in the De Vidovitch et al. [60] study comprised four blocks requiring associative capacities of increasing complexity. BPD participants performed significantly worse than the HCs for negative words which mainly consisted of self-descriptors, particularly during the two blocks with high cognitive demands. As mentioned above, the manipulation of self-concept coherence in Lowmaster’s [54] study resulted in more impulsive responses among high BP features participants compared to low BP features participants in the go/stop task. In the EST of Sieswerda, Arntz, Mertens, and Vertommen’s [66] study, self-descriptors related to stinginess did not produce more interference among the BPD participants compared to HCs but words related to negative BPD schema (powerless, unacceptable, and malevolent) did. In the EST of Sieswerda et al. [67] study, both schema-related words and schema unrelated words (self-descriptors of stinginess) induce more interference among BPD participants compared to HCs before a 3-year treatment. These results remained in nonrecovered BPD patients at the end of treatment but were completely reduced to normal for the recovered BPD group. In Arntz, Appels, and Sieswerda’s [64] study, words referring to a negative self-view produce more interference in the BPD group than in the HC group but did not produce more interference than other types of words (negative view of others, sexual abuse, and general negative words unrelated to BPD pathology). In the Scrambled Sentences Test used in Geiger et al.’s [73] study, participants were asked to rearrange six-word strings to create 5-word sentences beginning by “I…”. In this study, words were modified to include BPD specific contents. Authors reported that severity of the BP features was significantly correlated with the tendency to unscramble strings to create BPD-consistent sentences. Moreover, it seems that this tendency was accentuated when the task was completed under a cognitive load.

##### Stimuli or Procedures Related to BPD Material or Which Are Personally Relevant for the Participant

In their linguistic GNG, Silbersweig et al. [45] used words containing negative salient themes for BPD participants who displayed significant impairments during these negative no-go conditions compared to HCs. In the EST of Sieswerda, Arntz, Mertens, and Vertommen’s [66] study, words related to negative BPD schema (powerless, unacceptable, and malevolent) produced more interference among the BPD participants compared to HCs, whereas self-descriptors related to stinginess did not. In the EST of Sieswerda et al.’s [66] study, words of schema related as well as words of schema unrelated (self-descriptors of stinginess) both induced more interference among BPD participants compared to HCs before a 3-year treatment. These results remained in the nonrecovered BPD patients at the end of treatment, but were completely reduced to normal for the recovered BPD group. In the EST of Portella et al.’s [65] study, authors reported that the BPD group were slower to respond to the task than the HC group for BPD specific words. In the EST study of Sprock et al.’s [68] study, words which aimed to elicit common themes for BPD and MDD (anger and sadness words) did not produce more interference among BPD participants compared to HCs. In Wingenfeld et al.’s [69] study, words related to personal negative life events that were currently relevant induced more interference (compared to personal negative life events that were not currently relevant or general negative words) among BPD participants with a comorbid PTSD compared to the BPD group without PTSD and the HC group. In another study, Wingenfeld et al. [70] found that individuals with BPD had overall slower reaction times to the task regardless of the kind of emotional words (individual negative words or general negative words) compared to HCs. In their study, Winter et al. [71] reported that only the BPD group which experienced dissociation via an individual script driven imagery had slower reaction times for negative compared to neutral words. The BPD group who did not undergo dissociation did not perform differently than the HC group regarding the emotional words. Finally, Korfine and Hooley [48] reported that the BPD group recalled significantly more BPD-specific words among other types of words in a DFT task than did the HC group in the forget condition.

#### 3.2.2. Studies that Do Not Include Self-Reference Processes

This category included 15 studies totalizing 17 inhibition tasks (see Table 2). Among the eight tasks assessing PRI in absence of self-referential stimuli or processes, three resulted in a decrease in performance in BPD participants compared to that of HCs and five did not result in a group difference. Regarding the seven tasks assessing RDI in absence of self-referential stimuli or processes, three resulted in a decrease of performance among BPD participants compared to HCs, whereas four did not result in a group difference. Finally, among the two tasks assessing RPI in absence of self-referential stimuli or processes, none resulted in a decrease of performance among BPD participants compared to HCs.

##### Biological Procedures to Induce Stress or the Presence or Induction of Negative Emotional States

Carvahlo et al. [43] reported no group effect or significant interaction in their affective GNG task. All subjects reacted faster in the cortisol condition. In their passive avoidance learning task, Chapman et al. [44] found that participants with high BP features committed a greater number of impulsive responses than participants with low BP features and that participants with high BP features who were in a negative emotional state committed fewer impulsive responses than participants with high BP features who were in a low negative emotional state. In particular, fear, nervousness, and shame were negatively correlated with impulsivity among the group with high BP features. Two years later, this group conducted a study [42] in which participants were randomly assigned to a fear induction or a neutral mood induction. They found that participants with high BP features (but not low) committed a greater number of impulsive responses in the fear condition compared with the neutral condition.

##### The Mannheim Multicomponent Stress Test [80]

Cackowski et al. [31] reported a higher percentage of deficits in inhibition responses among BPD participants under the stress condition and not under the resting condition compared to HCs. Krause-Utz [46] replicated this finding with an IMT task. The BPD group performed significantly worse than the HC group only after the stress induction. However, in that same study, authors reported no difference between groups in the Go/Stop task following the stress condition.

##### Paced Auditory Serial Addition Task Computerized Version [81]

Krause-Utz [41] reported that regarding the IMT task, the BPD group showed better performance compared with patients in the BPD-ADHD group, ADHD group, and HC group under both conditions (stress and resting conditions). The authors reported no difference between groups for the DMT task.

##### General Emotional Words

General negative words used in the study of Arntz, Appels, and Sieswerda [64] produced more interference among the BPD group than the HC group but did not produce more interference than other types of words (negative view of others, negative self-descriptors, and referring to sexual abuse). The general negative words used in the EST of Domes et al.’s [49] study and of Portella et al.’s [65] study did not produce more interference than neutral words among BPD patients compared to HCs. The same absence of effect was observed with the general negative words used in the EST of Winter et al.’s [71] study in the absence of induced dissociation before the task, and with the general negative words that were not personally relevant which were used in the EST of Wingenfeld et al.’s [69] study. However, in the other EST of Wingenfeld et al.’s [70] study, general negative words induced interference as well as individual negative words among BPD participants compared to HCs. It was also the case in Mensebach et al.’s [74] study in which authors reported that BPD’s performance at the memory task was impaired when interference was obtained with general emotional words. Regarding the DFT, neither general negative words used in Domes et al.’s [49] study nor general negative words used in Korfine and Hooley’s [48] study produced impairments among the BPD group compared with the HC group.

## 4. Discussion

### 4.1. Summary of Objectives and Results

Impulsivity is a serious problem among BPD individuals. It is also a major diagnostic criterion and is considered to be central among neurobehavioral models of BPD. In order to better understand the nature of this symptom, recent studies have investigated the interaction between stress, negative emotion, and inhibition processes, and to date results are mixed.

The first aim of this systematic review was to verify if stress and negative emotional contexts or stimuli were able to disrupt BPD individuals’ performance on tasks measuring PRI, RDI, and RPI as defined by Friedman and Miyake [22]. The second aim of our study was to verify if self-referential stimuli or processes were able to impact on BPD individuals’ inhibition performance during stress and negative emotional contexts or stimuli, and if this impact was similar across inhibition processes. To do so, we categorized negative emotional or stressful stimuli and procedures according to Winter’s [50] criteria of BPD self-referential stimuli and to Lowmaster’s [54] procedure to stress the cohesion of the BPD self-concept.

Results of the present systematic review showed that the number of studies showing a decrease of performance in individuals with BPD compared to controls on tasks measuring PRI, RDI and RPI is very similar to the number of studies that did not find such a decreased performance (PRI: 9 vs. 8; RDI: 13 vs. 8; and RPI: 1 vs. 2). However, after having divided studies according to the presence or absence of self-referential stimuli or processes on the same tasks, results showed a clear trend. Among studies with self-referential stimuli or processes, the number of studies that found a decrease of performance in individuals with BPD compared to controls on tasks measuring PRI, RDI and RPI are twice as numerous than the number of studies that did not find such a decreased performance (PRI: 6 vs. 3; RDI: 10 vs. 4; and RPI: 1 vs. 0). Among studies without self-referential stimuli or processes, the number of studies that found a decrease of performance in individuals with BPD compared to controls on tasks measuring PRI, RDI and RPI are less numerous than the number of studies that did not find such a decreased performance (PRI: 3 vs. 5; RDI: 3 vs. 4; and RPI: 0 vs. 2).

However, several studies did not support our hypothesis. Regarding studies involving social stimuli, one could argue that stimuli or procedures were not personally relevant enough as they were selected from images depicting aversive scenes [61] or fearful faces [43] or from a procedure consisting listening to a non-relevant short story [33]. Similarly, since words used in Sprock, Rader, Kendall, and Yoder’s [68] study were specific for both BPD and depressive participants, they may not have induced a strong enough self-reference process. The study of Arntz, Appels and Sieswerda [64] did not find a specific bias for one of the four types of emotional words (negative view of others, negative self-descriptors, words referring to sexual abuse, and general negative words). It is possible that such a composition of various stimuli may have resulted in an ambiguous and arousing situation for BPD patients due to their weak sense of identity. Sieswerda, Arntz, Mertens, and Vertommen [66] reported that BPD participants showed a bias for BPD schema-related negative words but not for BPD schema-unrelated negative words, which consisted of self-descriptor words related to stinginess. In this study, participants were exposed additionally to BPD schema-related positive words and to BPD schema-unrelated positive words (self-descriptor words such as joyful). Consequently, it is possible to think that words related to stinginess may have induced less self-reference processes compared to the four types of words. The study of Sieswerda and Arntz [67] supported this hypothesis. Indeed, in the latter study, participants were exposed only to negative BPD schema-related words and to words related to stinginess, and results showed that BPD participants had a bias for both types of words. General negative words used in other studies [70,74] led to impaired inhibition performance. However, without the exhaustive list of words, it is difficult to verify if these words contained some self-referential words. The study of Wingenfeld [69] seemed to highlight the importance of comorbid PTSD. The stress induced by the MMST [80] in Cackowski et al.’s [31] study and by the IMT task of Krause-Utz et al.’s [46] study lead to impairments in the performance of BPD group in accordance with Linehan’s affective dysregulation hypothesis. However, this was not the case for the go/stop task of the same study [46]. Finally, the fear induction used in Chapman, Dixon-Gordon, Layden, and Walters’s [42] study seemed to have led participants with high BP features to commit a greater number of impulsive responses. Since the procedure involved watching a chase scene between a serial killer and a police woman, it may be argued that this procedure could have contained trauma related information and consequently contained a self-reference process for this group.

In a nutshell, results of the present systematic review indicated that when stimuli or procedures used to induce a stress or negative emotion involve self-referential stimuli or processes before or during the inhibition task, BPD individuals’ inhibition performance seems to be more disrupted than when stimuli or procedures used to induce a stress or negative emotion does not involve self-referential stimuli or processes, and this seems to apply to PRI, RDI, and RPI. The present systematic review supported Winter’s hypothesis that higher distraction towards negative emotional stimuli in BPD individuals compared to HCs could be explained by the fact that negative stimuli are more self-relevant for the BPD individuals due to their negative self-concept. Consequently, these stimuli seem to influence BPD individuals’ performance in tasks assessing RDI. Moreover, the present review extended Winter’s hypothesis to the two other inhibition processes—PRI and RPI.

### 4.2. Proposition of A Model to Integrate the Self-Concept and Inhibition Processes

We believe that Winter’s model can be meaningfully integrated with Kernberg’s model in order to elucidate the complex relation between inhibition processes and the self-concept. We view Kernberg’s model as compatible with Winter’s model in the sense that both models propose a feedback loop between long-term memory structure such as self-concept and active processes of emotion regulation. In Winter’s model, the BPD attentional filter will influence the negative self-concept that is encoded and stored in long-term memory and, in turn, the stored negative concept will influence the information that is filtered. In Kernberg’s model, which is more inseparable from social reality, the feedback loop extends the individual’s cognitive–affective structures and processes to reach the external other in the social realm. Indeed, the BPD individual will use splitting after a social frustration, resulting in a disavowed negative self-representation perceived and attributed to the external other. In turn, the reaction of the external other towards the BPD individual’s emotion and behavior will be used by the latter to encode and store the negative representation of the self-concept (which is indissociably related to the concept of the other).

A recent study by Gagnon, Vintiloiu, and McDuff [82] has shown that it is possible to empirically study the unique contribution of the self-concept (emotionally related with the concept of the other) and the splitting on BPD impulsive behaviors as conceptualized in Kernberg’s model. Moreover, each personality dimension was shown to be related to a specific aspect of BPD impulsivity. The BPD self-concept was associated with the borderline impulsivity trait (i.e., the tendency to act in a self-destructive way). This result was interpreted as the mutual influence between self-concept, cognitive processes, and effortful control [83]. According to this view, cognition contributes to personality by providing the representational aspects of affect activation. The more complex the representations, the more complex and nuanced the emotional experiences. Splitting, on its part, was associated with the frequency of impulsive behaviors in real life. This result was interpreted as the influence of splitting on state impulsivity (i.e., a negative emotional state associated with a temporary lack of impulse control). Given that splitting is a rigid and inflexible way to cope with tension arising from conflicts, it is associated with sudden changes in the way people are perceived leading to mood changes and affect instability [84]. Also, specific to splitting, there is a lack of emotional contact between that part of the individual’s personality and the rest of the self-experience (the other split representations of the conflict), thus depriving the individual from having access to crucial information which would allow them to self-regulate their behaviors.

**Figure 2:**
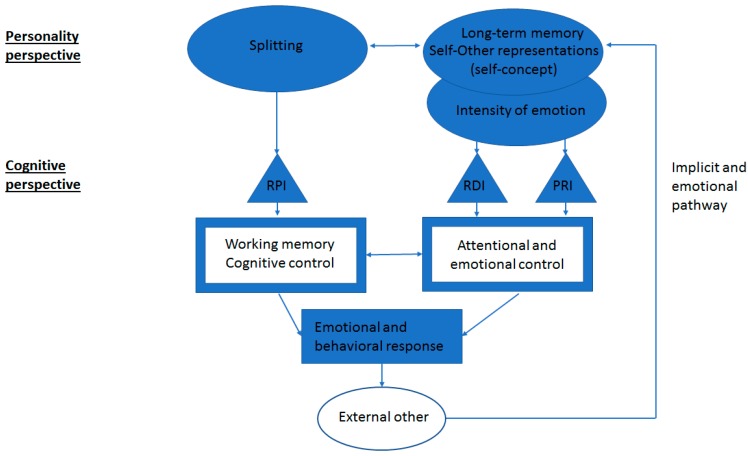
This model integrates the self-concept and inhibition processes in BPD. From the personality perspective, the self-concept, which belongs to long-term memory, is composed of self-representations and other-representations linked by an affect tone. The polarized positive or negative segments of self- and other-representations in BPD is associated with more intense and less nuanced emotions, which in turn interact with attentional and emotional control. The nonaffectively integrated BPD self-concept maintains a functional relationship with the use of splitting, which is a defense mechanism that separates negative self- and other-representations from positive self- and other-representations. The splitting belongs to working memory as a cognitive control process. From the cognitive perspective, each inhibition process plays a respective role in the regulation of cognitive, attentional and emotional control processes. In reaction to the individual’s impulsive and negative emotional and behavioral response, the other person’s (external other) negative response (i.e., social reject) reinforces the negative self-concept through implicit and emotional pathways of communication.

In order to explain the effect of self-referential stimuli and processes in BPD inhibition deficits, we propose a model of BPD impulsivity in order to integrate Winter’s and Kernberg’s models in the following way (see Figure 2):

From the personality perspective, the BPD self-concept is viewed as composed of self- and other-representations which are polarized in positive and negative segments. Also, the self-concept is overlapping with the intensity of emotion as both depend on the complexity of self- and other-representations. The lack of affective integration in the BPD self-concept is closely linked with splitting as both reinforce each other. From the cognitive perspective, these personality processes may have preferential associations with cognitive processes. Following a perceived social rejection, splitting may be associated with a reduced cognitive control via a lack of inhibition of the more negative cognitions in such a way that it prevents the individual to have access to the more positive cognitions. For its part, the BPD self-concept, through its overlapping with emotional intensity, may be associated with reduced attentional and motor controls as both are influenced by a lack of emotional regulation and a need to discharge the negative emotional tension through impulsive behaviors. These control processes would be associated with specific inhibition processes in the expression of the BPD individual’s emotional response—the RPI with the regulation of cognitive control and both RDI and PRI, which are functionally related [18] with the regulation of attentional and motor controls. The BPD individual’s emotional response leads the external other to react in a negative way that will be encoded and stored in the self-concept through an implicit and emotional pathway of communication. A recent study brought partial support to this model by showing that perceived social rejection vs. inclusion had a significant effect on RPI performance as a function of the participants’ frequency of use of splitting [85]. Other studies supported the existence of the model’s implicit and emotional pathway, by showing an association between an implicit measure of the BPD self-concept with a projective instrument and BDP impulsivity [86], as well as an association between an implicit measure of hostile cognitions or concept of self with event-related potentials and aggression [87,88,89]. More studies are in need to verify this model and particularly if the BPD self-concept and splitting dimensions of personality can be specifically related to inhibition processes. Such research could also inform us about the direction of the association between inhibition processes and personality dimensions. For example, pre-post-treatment studies could reveal that progress in the complexity of the self-concept results in improvement in the performance on RDI and PRI tasks among BPD participants.

### 4.3. Limitations of the Present Study

Overall, the results of the present systematic review must be taken with caution since confounding factors which have been described in the literature may have influenced the inhibition performances in the reported studies such as comorbidities, particularly with major depressive disorder, PTSD and ADHD, dissociative symptoms, the intensity of overall BPD symptoms, and the presence of psychotherapy and medication [17,50,52]. Indeed, most studies reported in the present review did not control for the influence of these factors, particularly comorbidities with PTSD, ADHD, and dissociative symptoms. Most studies had samples composed of severe BPD patients limiting the generalization of observations to less severe patients or BPD high features individuals. Moreover, only a few studies reported information regarding the psychological and pharmacological treatment status of participants.

In addition, differences between inhibition tasks across studies may explain some part of the heterogeneous evidence. Another limit was that the exhaustive list of stimuli used in tasks, particularly words used in the EST studies, were not always available to guide the categorization of stimuli as self-referential. In these cases, we based our decision on the description given by the authors.

The literature in this area is highly heterogeneous both among experimental stimuli and participant characteristics. Thus, comparing studies in this field is enormously challenging. Another challenge that researchers face in this area is to better understand the role of emotional, situational and self-concept variables in impulse control deficits in BPD [14]. Indeed, deficits of inhibition are modulated by emotional states or the current relevance of negative stimuli for the self-concept. Given that inhibition processes are composed of several functions, future studies should try to better differentiate which emotion contributes to disrupt which component of inhibition in which situation. For example, it has been shown that fear can hamper the inhibition of a previously punished response [42]. What can be said about other borderline-related feelings such as anger, abandonment, rage, sadness, and anxiety? Do these emotions have the same effect on PRI, RDI, and RPI in various social situations? Otherwise, inhibition processes should be investigated in relation to different components of the BPD self-concept. For example, self-concept-related variables, such as self-representations, other-representations, personal schemas, attributional bias, or defensive styles, can also interact with inhibition processes in the emergence of impulsive behaviors. It would be important to include such personality variables in future studies on inhibition.

## 5. Conclusions

To the best of our knowledge, this is the first systematic review regarding the role of self-referential stimuli and processes in modulating the influence of stress and emotional stimuli and procedures on BPD performance on PRI, RDI, and RPI tasks. This review has taken into consideration three specific inhibition processes measured by behavioral tasks and has categorized the various negative emotional stimuli and procedures called upon to induce a stress or emotional state in participants. All the tasks in the studies were selected and categorized based on clear definitions and criteria, and all ambiguous decisions were resolved with inter-rater agreement.

Overall, the present systematic review highlights the importance of taking into consideration self-referential stimuli and processes in the investigation of the influence of stress and negative emotion on BPD individuals’ inhibition deficits in order to better understand the nature of their impulsivity. A theoretical model was proposed to integrate both personality dimension related to the self-concept and inhibition processes with the aim of guiding future empirical research.

## Figures and Tables

**Table 1:** Results of 19 studies with self-reference processes during the emotional or stressful condition.

First Author, Year	Participants	Brief Description of the Experiment (Inhibition Process *)	Brief Description of the Stress or Negative Emotion **	Decreased Performance in BPD Group? ***
Carvahlo, 2013 [43]	32 BPD	An affective GNG (PRI)	Fearful faces	NO
	32 HC			
Jacob, 2013 [33]	17 BPD	A GNG (PRI)	Short stories to induce anger of social nature	NO
	18HC			
Ernst, 2018 [52]	20 MDD	A GNG (PRI)	Social exclusion condition of Cyberball	YES
	22 BPD-MDD			
	22 HC			
De Vidovich, 2016 [60]	8 BPD	An affective GNG (PRI)	Negative words (mainly self-descriptors)	YES
	9 HC			
Silbersweig, 2007 [45]	16 BPD	A linguistic GNG (PRI)	Negative words reflecting salient themes for BPD	YES
	14 HC			
Lowmaster, 2014 [54]	398 undergraduates with high and low BP features	Go/Stop task (PRI)	A manipulation of self-concept coherence	YES
Bertsch, 2018 [56]	30 BPD	An approach-avoidant task (PRI)	Angry faces	YES
	28 HC			
Paret, 2016 [61]	21 BPD	A learning task with emotional interference (PRI)	Images from database depicting aversive scenes with human beings	NO
	15 HC			
Dixon-Gordon, 2017 [57]	17 BPD	Reinforcement learning task (PRI)	A personal narrative of a distressing interpersonal interaction	YES
	20 past-year/mood anxiety disorder			
	23 HC			
Arntz, 2000 [64]	15 BPD 12 Cluster C PD 15 HC	An EST during subliminal and supraliminal conditions (RDI)	Words reflecting negative view of others	YES
			Words reflecting sexual abuse	YES
			Words reflecting negative self-view	YES
Portella, 2011 [65]	38 BPD	An EST (RDI)	Borderline negative words	YES
	23 HC			
Sieswerda, 2007 [66]	16 BPD 18 Cluster C PD 16 axe 1 disorder 16 HC	An EST (RDI)	BPD schema-related negative words	YES
			BPD schema-unrelated negative words (self-descriptor stinginess)	NO
Sieswerda, 2007 [67]	24 BPD patients 16 BPD nonpatients 23 HC	An EST (RDI)	BPD schema-related negative words	YES
			BPD schema-unrelated negative words (self-descriptor stinginess)	YES
Sprock, 2000 [68]	18 BPD	An EST (RDI)	Words common for BPD and MDD (anger and sadness)	NO
	17MDD			
	16HC			
Wingenfeld, 2009 [69]	31 BPD	An EST (RDI)	Words related to personal negative events that were currently relevant Words related to personal negative events that were not currently relevant	NO (BPD-PTSD)
	49 HC			NO
Wingenfeld, 2009 [70]	20 BPD	An EST (RDI)	Individual negative words	YES
	20 HC			
Winter, 2015 [71]	40 BPD	An EST (RDI)	Induction of dissociation (via personal script driven imagery before the task) + negative words	YES
	20 HC			
Geiger, 2014 [73]	194 undergraduates (19% high BP features)	A Scrambled Sentences Test (RPI)	Sentences which resemble BPD’s cognitive distortions	YES
Korfine, 2000 [48]	23 BPD	A DFT (RPI)	BPD type words	YES
	20 HC			

*, PRI = prepotent response inhibition; RDI = resistance to distractor interference; RPI = resistance to proactive interference; **, which are compared to neutral or positive stimuli or procedures in every study; ***, Significant decrease of performance in the BPD group compared to the HC group on behavioral measures of inhibition capacities in a stress or negative emotional condition.

**Table 2:** Results of 15 studies without self-reference processes during the emotional or stressful condition.

First Author, Year	Participants	Brief Description of the Experiment (Inhibition Process *)	Brief Description of the Stress or Negative Emotion **	Decreased Performance in BPD Group? ***
Carvahlo, 2013 [43]	32 BPD 32 HC	An adapted affective GNG (PRI)	10 mg of hydrocortisone before the task	NO
Cackowski, 2014 [31]	31 BPD 30 HC	A Go/Stop task (PRI)	The MMST	YES
Krause-Utz, 2016 [46]	30 BPD 28 ADHD 32 HC	A Go/Stop task (PRI)	The MMST	NO
Krause-Utz, 2013 [41]	15 BPD 15 BPD-ADHD 15 ADHD 15 HC	An IMT (PRI)	The PASAT-C	NO (better)
		A DMT (PRI)	The PASAT-C	NO
Krause-Utz, 2016 [46]		An IMT (PRI)	The MMST	YES
Chapman, 2008 [44]	39 high BP features 56 low BP features	A passive avoidance learning task (PRI)	The self-reported negative affective state	NO (better)
Chapman, 2010 [42]	28 high BP features 44 low BP features	A passive avoidance learning task (PRI)	Fear induction: to watch a chase scene between a serial killer and a police woman	YES
Arntz, 2000 [64]	15 BPD 12 Cluster C personality disorder (PD) 15 HC	An EST during subliminal and supraliminal conditions (RDI)	General negative words	YES
Domes, 2006 [49]	28 BPD 30 HC	An EST (RDI)	Negative words	NO
Portella, 2011 [65]	38 BPD 23 HC	An EST (RDI)	Negative words	NO
Wingenfeld, 2009 [69]	31 BPD 49 HC	An EST (RDI)	Negative words that were not personally relevant	NO
Wingenfeld, 2009 [70]	20 BPD 20 HC	An EST (RDI)	General negative words	YES
Winter, 2015 [71]	40 BPD 20 HC	An EST (RDI)	Negative words	NO
Mensebach, 2009 [74]	47 BPD 70 HC	A verbal memory task during interference learning condition (RDI)	Negative words	YES
Korfine, 2000 [48]	23 BPD 20 HC	A DFT (RPI)	Negative words	NO
Domes, 2006 [49]		A DFT (RPI)	Negative words	NO

*, PRI = prepotent response inhibition; RDI = resistance to distractor interference; RPI = resistance to proactive interference; **, which are compared to neutral or positive stimuli or procedures in every study; ***, Significant decrease of performance in the BPD group compared to the HC group on behavioral measures of inhibition capacities in a stress or negative emotional condition.
